# Congenital hypopituitarism in two brothers with a duplication of the ‘acrogigantism gene’ *GPR101*: clinical findings and review of the literature

**DOI:** 10.1007/s11102-020-01101-8

**Published:** 2020-11-13

**Authors:** Melitza S. M. Elizabeth, Annemieke J. M. H. Verkerk, Anita C. S. Hokken-Koelega, Joost A. M. Verlouw, Jesús Argente, Roland Pfaeffle, Sebastian J. C. M. M. Neggers, Jenny A. Visser, Laura C. G. de Graaff

**Affiliations:** 1 Department of Internal Medicine, Section of Endocrinology, Erasmus MC, University Medical Center Rotterdam, Rotterdam, The Netherlands; 2 Department of Pediatrics, Subdiv. Endocrinology, Erasmus MC Rotterdam, Rotterdam, The Netherlands; 3 Department of Endocrinology, Fundación Investigación Biomédica del Hospital Infantil Universitario Niño Jesús, Instituto de Investigación Biomédica la Princesa, Madrid, Spain; 4 Hospital for Children and Adolescents, University of Leipzig, Leipzig, Germany; 5 Dutch Growth Research Foundation, Rotterdam, The Netherlands; 6 Academic Center for Rare Growth Disorders, Erasmus MC Rotterdam, Rotterdam, The Netherlands; 7 Centro de Investigación Biomédica en Red Fisiología de la Obesidad y Nutrición (CIBEROBN), Madrid, Spain; 8 IMDEA Food Institute, Campus of International Excellence (CEI) UAM + CSIC, Madrid, Spain; 9 Department of Pediatrics, University Autonoma de Madrid, Madrid, Spain

**Keywords:** Pituitary gland, Transcription factors, Gene duplication, Acromegaly, G-protein coupled receptor

## Abstract

**Purpose:**

Congenital hypopituitarism (CH) can cause significant morbidity or even mortality. In the majority of patients, the etiology of CH is unknown. Understanding the etiology of CH is important for anticipation of clinical problems and for genetic counselling. Our previous studies showed that only a small proportion of cases have mutations in the known ‘CH genes’. In the current project, we present the results of SNP array based copy number variant analysis in a family with unexplained congenital hypopituitarism.

**Methods:**

DNA samples of two affected brothers with idiopathic CH and their mother were simultaneously analyzed by SNP arrays for copy number variant analysis and Whole Exome Sequencing (WES) for mutation screening. DNA of the father was not available.

**Results:**

We found a 6 Mb duplication including *GPR101* and *SOX3* on the X-chromosome (Xq26.2-q27.1) in the two siblings and their mother, leading to 2 copies of this region in the affected boys and 3 copies in the mother. Duplications of *GPR101* are associated with X-linked acrogigantism (the phenotypic ‘opposite’ of the affected brothers), whereas alterations in *SOX3* are associated with X-linked hypopituitarism.

**Conclusion:**

In our patients with hypopituitarism we found a 6 Mb duplication which includes *GPR101*, a gene associated with X- linked gigantism, and *SOX3*, a gene involved in early pituitary organogenesis that is associated with variable degrees of hypopituitarism. Our findings show that in duplications containing both *GPR101* and *SOX3*, the growth hormone deficiency phenotype is dominant. This suggests that, if *GPR101* is duplicated, it might not be expressed phenotypically when early patterning of the embryonic pituitary is affected due to *SOX3* duplication. These results, together with the review of the literature, shed a new light on the role of *GPR101* and *SOX3* in pituitary function.

**Electronic supplementary material:**

The online version of this article (10.1007/s11102-020-01101-8) contains supplementary material, which is available to authorized users.

## Introduction

Normal development and function of the pituitary gland is crucial for several important physiological processes in the human body, such as growth, reproduction, lactation, response to stress, blood pressure, energy management and metabolism [1, 2]. Congenital hypopituitarism (CH) is a rare disorder with an estimated incidence of 1:3000–1:4000 live births. It is characterized by the diminished production or secretion of one or more of the pituitary hormones [3, 4].

Growth hormone deficiency (GHD) is the most common form of hypopituitarism. Both children and adults with GHD may present with short stature, increased fat mass and decreased lean body mass, delayed skeletal maturation, truncal obesity, abnormal glucose and lipid metabolism and an increased risk of cardiovascular disease [3, 4]. GHD can either be isolated (IGHD) or combined with other pituitary hormone deficiencies (CPHD). CPHD is defined as any combination of two or more pituitary hormone deficiencies, whereas in *pan*hypopituitarism all pituitary hormones are deficient [5].

The vast majority of GHD cases are idiopathic. Up to 30% of cases are familial, which suggests a genetic etiology [6, 7]. As a result of dedicated genetic studies, such as the Dutch HYPOPIT study, our knowledge about the genetic etiology of GHD has drastically improved. Frequent causes of IGHD are mutations in the Growth Hormone 1 *(GH1)* gene and the Growth Hormone Releasing Hormone Receptor (*GHRHR)* gene [4, 7]. In Dutch CPHD patients we have previously studied several known disease related genes encoding pituitary transcription factors, such as *PROP1* [MIM 601,538), *HESX1* (MIM 601802), *POU1F1* (MIM 173110), *LHX3* (MIM 600577)*, LHX4* (MIM 602146), *OTX2* (MIM 600037), *SHH* (MIM 600725), *HHIP* (MIM 606178) and *GLI2* (MIM 165230). However, we found a genetic explanation in only 10% of the patients, leaving the majority of cases unsolved [8–12]. When candidate gene analysis has turned out negative, array based copy number variation analysis and Next Generation Sequencing (NGS) is a next step. In this study we present the surprising results of NGS in two brothers with idiopathic CH.

## Material and methods

### Genetic analysis

#### DNA isolation

Genomic DNA of the two brothers and their mother was extracted from peripheral whole blood samples according to standard procedures. The samples were subsequently analyzed by SNP array and Whole Exome Sequencing (WES).

#### Copy number variant analysis by SNP array

DNA was hybridized to Illumina Human CytoSNP850K SNP arrays according to standard protocol. Copy number analysis was performed using Nexus 8.0 from BioDiscovery.

#### WES

Genomic DNA was fragmented into 200–400 base pairs (bp) fragments using Covaris Adaptive Focused Acoustics shearing according to the manufacturer’s instructions (Covaris, Inc., Woburn, MA). Illumina TruSeq DNA Library preparation (Illumina, Inc., San Diego, CA) was performed on a Caliper Sciclone NGS workstation (Caliper Life Sciences, Hopkinton, MA), followed by exome capture using the Nimblegen SeqCap EZ V2 kit (Roche Nimblegen, Inc., Madison, WI). This capture targets 44 Mb of exonic regions covering 30,246 coding genes, 329,028 exons and 710 miRNAs. Paired-end 2 × 100 bp sequencing was performed at 6 samples per lane on Illumina HiSeq2000 sequencer using Illumina TruSeq V3, resulting in 6 Gb of sequencing data.

### Literature search

In order to explain the phenotype of the two brothers, we performed an extensive literature search of all genes included in the duplicated region using OMIM, NCBI, MGI and Pubmed online databases. PubMed search was carried out using the names of all duplicated genes, combined with the terms [‘hypopituitarism’ OR ‘growth retardation’ OR ‘growth hormone deficiency’ OR ‘growth hormone’ OR ‘combined pituitary hormone deficiency’] AND ‘congenital’.

## Results

### Clinical data

The index case, an Italian male with idiopathic CH, presented with growth retardation late in life, with a height SDS of—2.1 at the age of 16 years. BMI was normal. The GH peak during a GH stimulation test was 2.16 µg/L (ref > 6.66 µg/L). Apart from GHD, he was diagnosed with central hypothyroidism, hypogonadism and hypocortisolism. Magnetic resonance imaging (MRI) revealed anterior pituitary hypoplasia (APH) and an ectopic posterior pituitary (EPP). His brother also had growth retardation. Despite the fact that he did not present until the age of 21 years, he had always been small (height—3 SDS). The GH peak during his GH test was 1.86 µg/L. Although the original laboratory values and MRI images of both brothers were not available, the medical files reported that all pituitary hormone concentrations as well as IGF-I and IGFBP3 were low. Both brothers had normal cognition and no other birth defects. The unaffected mother had no pituitary hormone deficiencies and a height of 153.5 cm (− 1.5 SDS). Clinical data of the father were not available.

### Genetic analysis

**Fig. 1:**
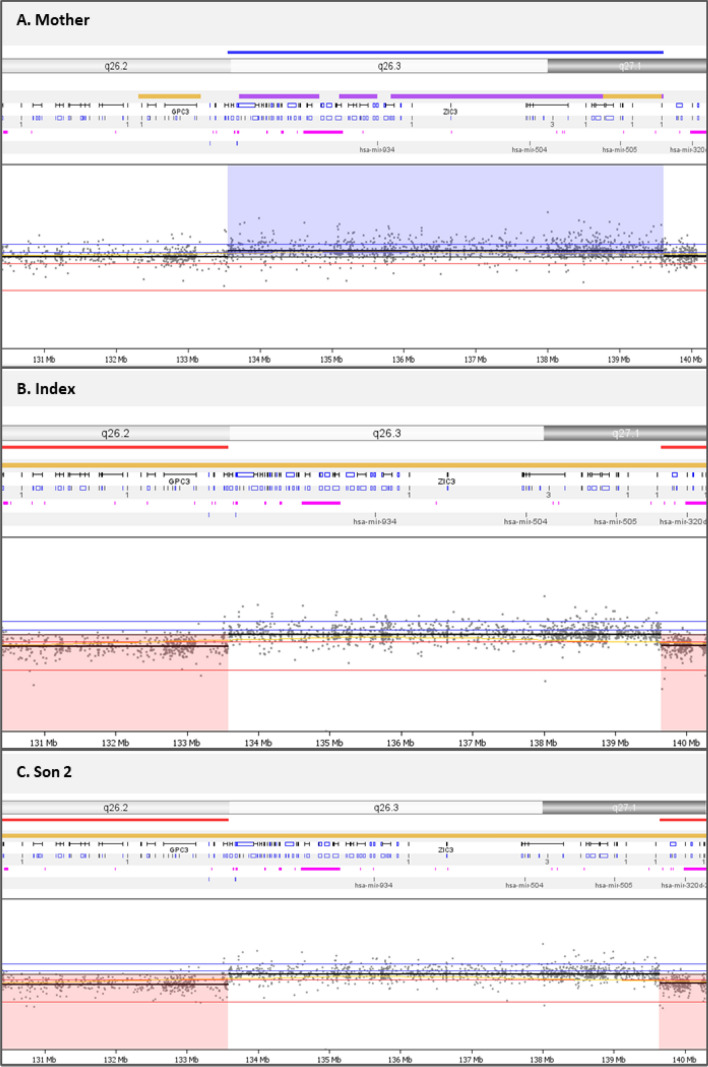
SNP array results of the two brothers and their mother. LogR ratios and B-allele frequencies (BAF) are indicated. Duplications are shown between vertical bars for **a** the mother, **b** and **c** the affected brothers

**Fig. 2:**
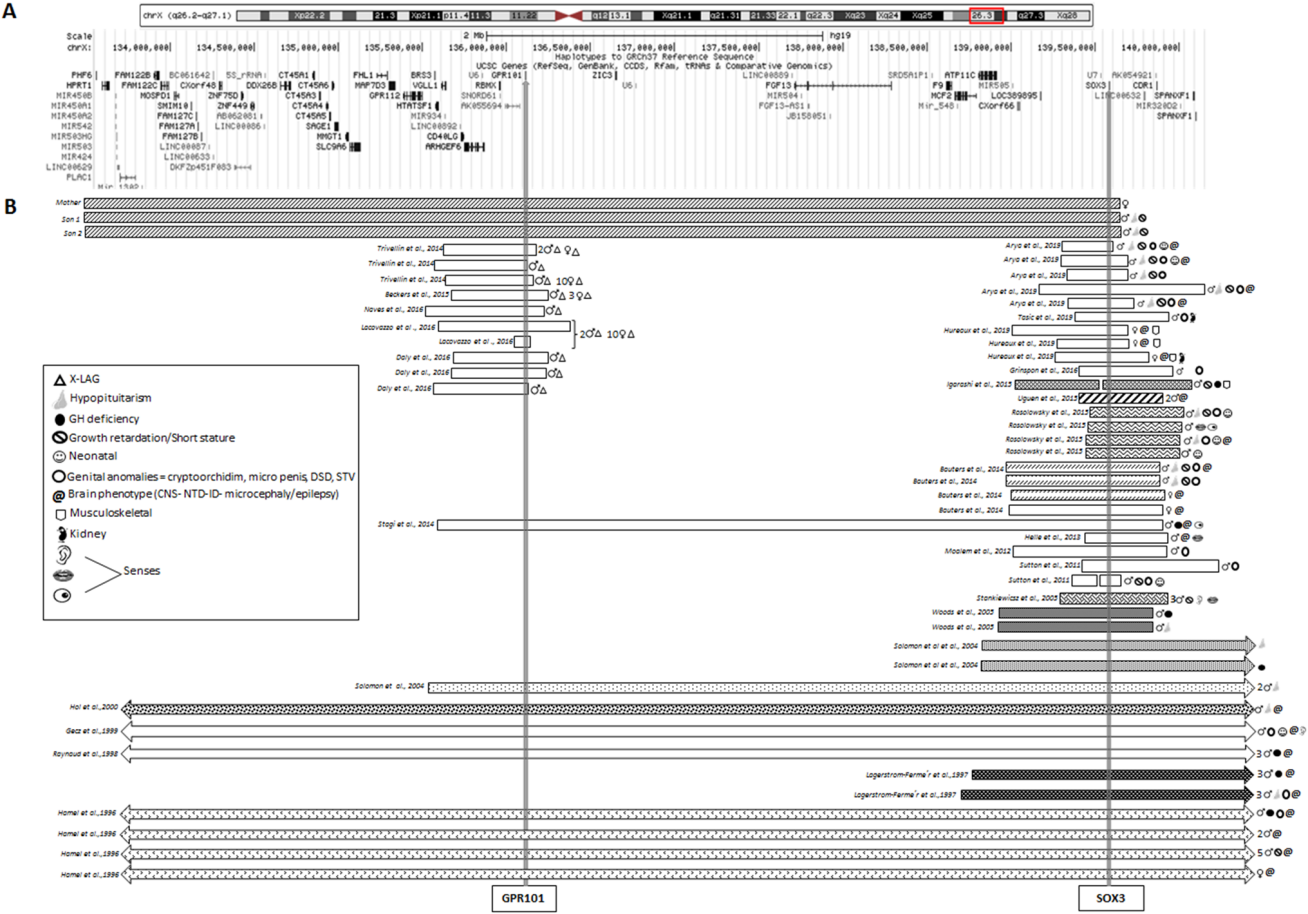
**a** Overview of 70 genes included in the duplicated region. **b** All duplications and deletions found in the literature involving *GPR101* and *SOX3*

SNP array data analysis revealed a 6 Mb duplication of chromosome Xq26.2-q27.1 in all 3 subjects. Figure 1 shows the 6 Mb duplication that results in two copies of part of the X-chromosome in the affected brothers and 3 copies in the mother. The duplicated region (chrX: 133.553.751–139,613,851; build 37) contains 70 genes (Fig. 2a). Table 1 shows the phenotypes associated with defects in these genes. The duplicated region includes *GPR101* (MIM 300393), a gene previously described in patients with X-linked acrogigantism (X-LAG) and acromegaly, which is the opposite clinical phenotype of our patients.

**Table 1:** Genes included in the duplicated region of the two brothers, clustered by disease association

	Gene symbol	Gene description	Disease association
Acromegaly Gigantism	GPR101	G protein-coupled receptor 101	X-linked acro-gigantism (X-LAG),pituitary adenoma, excessive GH secretion and rapid growth beginning in early childhood
Short stature	MOSPD1	Motile sperm domain containing protein 1	Short stature and abnormal right ventricle development
Cancer	LINC00629 CXorf48	Long intergenic non-protein coding RNA 629 Chromosome X open reading frame 48	Gastric cancer Chronic myeloid leucemia
	LINC00086	Long intergenic non-protein coding RNA 86	Gastric cancer
	CT45A1	Cancer/testis antigen family 45, member A1	
	CT45A3	Cancer/testis antigen family 45, member A3	
	CT45A4	Cancer/testis antigen family 45, member A4	
	CT45A5	Cancer/testis antigen family 45, member A5	
	CT45A6	Cancer/testis antigen family 45, member A6	
	*SAGE1*	Sarcoma antigen 1	Glioma and urothelial Cancer
	VGLL1	Vestigial like 1 (Drosophila)	
Intellectual disability	*PHF6*	PHD finger protein	Borjeson-Forssman-Lehmann syndrome (BFLS), a disorder characterized by intellectual disability (ID), epilepsy, hypogonadism, hypometabolism, obesity, swelling of subcutaneous tissue of the face, narrow palpebral fissures, and large ears
	FGF13	Fibroblast growth factor 13	Borjeson-Forssman-Lehmann syndrome (BFLS)
	SLC9A6	Solute carrier family 9, subfamily A	Intellectual disability (ID), X-linked syndromic cognitive disability, Christianson type
	RBMX	RNA binding motif protein, X-linked	X-linked intellectual disability syndrome
	HPRT1	Hypoxanthine phosphoribosyltransferase 1	Lesch-Nyhan syndrome (neurological and behavioural abnormalities and the overproduction of uric acid)
	*SOX3*	SRY (sex determining region Y)-box 3	X-linked intellectual disability (ID) with growth hormone deficiency (GDH), X-linked hypopituitarism, 46,XX, Disorder of Sex Development (DSD) and neural tube defects (NTD)
Reproduction	MIR503HG	MIR503 host gene (non-protein coding)	Pre-Eclampsia
	*PLAC1*	Placenta-specific 1 (PLAC1)	Pre-Eclampsia
	Mir_1302	Rfam model RF00951 hit found at contig region AL672032.6/126123-126174	Recurrent embryo implantation failure (RIF), male infertility
Hematologic diseases	F9	Coagulation factor IX (F9)	Factor IX deficiency, also called haemophilia B or Christmas disease
	*ATP11C*	ATPase, class VI, type 11C	Congenital haemolytic anaemia
Other	*MIR503*	microRNA 503	Tumor suppression
	CD40LG	CD40 ligand (CD40LG)	Hyper-IgM syndrome
	*ZIC3*	Zic family member 3	Heterotaxy and congenital heart disease
	*DDX26B*	DEAD/H box polypeptide 26B	Autism spectrum disorder
	*FHL1*	Four and a half LIM domains 1	Emery-Dreifuss muscular dystrophy, Reducing body myopathy (RBM), Uruguay faciocardiomusculoskeletal syndrome

The duplication also includes *SOX3* (MIM 313430), a gene associated with variable degrees of X-linked hypopituitarism and GHD, sometimes combined with intellectual disability (ID). Additional information of all genes located in the duplicated region is documented in Supplementary Table 1. WES data of the brothers and their mother returned negative.

### Literature search

*GPR101* and *SOX3* duplications and deletions published to date, with the associated clinical findings are shown in Fig. 2b. Of all previously reported X-LAG cases, 68% were females with germline duplications and 31% were males carrying somatic duplications. For *SOX3* duplications, there was a male predominance with 89.1% males and 8.9% females, respectively. *SOX3* duplications were associated with variable phenotypes, ranging from hypopituitarism, GHD only, intellectual disability (ID), neural tube defect (NTD), Disorders of Sex Development (DSD), or a combination of these phenotypes (Fig. 2b). Of the reported males with *SOX3* duplications, 42% had hypopituitarism, 28% had GHD and 48% had ID. Among males with duplications that included *SOX-3*, DSD was present in 32% and NTD in 8%. Eighty percent of affected females had a NTD, of which 60% were fetuses of terminated pregnancies. Sixty-nine percent of the described *SOX3* cases were of familial origin, where the carrier mother transmitted the duplication to the affected offspring. Table 2 shows the endocrine phenotypes that have been reported in the literature in patients with *SOX3* duplications. Table 3 shows non-endocrine features associated with *SOX3* duplications. Although less frequent, *SOX3* point mutations and poly alanine tract mutations have also been reported. Pituitary MRI findings and hormone deficiencies associated with these mutations are shown in Table 4.

**Table 2:** Endocrine phenotypes of *SOX3* duplications

Refs	Case	Sex	Growth	Gonads	Hormone deficiencies	Neonatal	MRI
[38]	F1	F			NA		Chiari II malformation
[38]	F2	F			NA		Chiari II malformation, voluminous AP, absent PP
[38]	F3	F			NA		Chiari II malformation
[39]	F IV	?					Hydrocephalus
[39]	F	F					hydrocephalus
[40]	III.2	F	SS/GR				
[40]	II.2	F	SS/GR				
[40]	III.4	F	SS/GR				
[32]	I	M	FTT	Micropenis, hypoplastic scrotum	GH, LH/FSH, TSH, ACTH	Jaundice, hypoglycemia	APH, EPP
[32]	II	M	SS/GR	Micropenis and STV	GH, ACTH, TSH, LH/FSH	Hypoglycemia	ACC, hydrocephalus
[32]	III	M	SS/GR	Slender phallus and STV	LH/FSH		Thin CC, hydrocephalus
[32]	IV	M	SS/GR		GH, TSH		APH, EPP
[32]	V	M	SS/GR	Micropenis and STV, hypogonadism, pubertal delay	GH, LH/FSH		Partial ACC, absent SP, heterotopic grey matter
[21]		M		STV and coronal hypospadias	NA		
[41]		M		Hypospadias, cryptorchidism, ovarian tissue and primary follicles	Testosterone, AMH		
[42]	Index	M	Mild SS/GR		mild GH		
[43]	F2	M					
[43]	F3	M					
[44]	C1	M	SS/GR	Microphallus and undescended testes	Hypopituitarism,GH, testosterone	Hypoglycemia, Jaundice	CVP, shallow pituitary fossa
[44]	C2	M					Thin CC, poorly developed pituitary gland
[44]	C3	M		Microphallus, small penis, undescended testis, underdeveloped scrotum	All including diabetes insipidus	Hypoglycemia	Absent pituitary gland and stalk
[44]	C4	M				Hypoglycemia,	APH, hypoplastic pituitary stalk
[37]		M			GH		
[39]	III.7	M	Growth delay	Undescended testicle, pubertal delay	GH, LH/ FSH		
[39]	III.1	M	SS	Small testicles	GH, LH/ FSH		Temporal brain atrophy
[45]		M					
[46]		M		Bifid scrotum and penoscrotal hypospadias			
[47]	A	M		SRY, 46, XX negative			
[24]	2	M			GH, TSH, ACTH, LH/FSH		APH, undescended PP, absent infundibulum
[24]	1	M			GH		APH, undescended hypoplastic infundibulum
[28]	A II.1	M			GH, TSH		
[28]	A II.2	M			GH		
[28]	B II.1	M			GH, TSH		
[28]	B II.2	M			GH, TSH		
[48]		M		Cryptorchidism with a small penis, hypogonadism		Hypoglycaemia Microcephaly	
[30]		M			Pan-hypopituitarism		
[29]	1	M			GH		
[29]	2	M			GH		
[29]	3	M			GH		
[27]	IV	M			GH, NA		
[27]	IV.4	M			GH		
[27]	IV.5	M			GH		
[27]	II.5	M		Hypogonadal	GH, TSH, PRL		
[27]	III.3	M			GH, TSH, PRL		
[27]	III.9	M		Hypogonadal	GH, TSH, PRL		
[31]	III.9	M		Mild gynaecomastia	GH		
[31]	II.6	M			NA		
[31]	II.7	M			NA		
[31]	II.8	M	SS				
[31]	III.3	M	GR				
[31]	III.6	M	SS				
[31]	III.7	M	SS				
[31]	IV.6	M	SS				
Present case		M	SS/GR		All pituitary hormones		APH, EPP
Present case		M	SS/GR		All pituitary hormones		NA

*AP* anterior pituitary, *APH* anterior pituitary hypoplasia, *EPP* ectopic posterior pituitary, *EP* ectopic pituitary, *NA* not assessed, *ACC* agenesis corpus callosum, *CC* corpus callosum, *SP* septum pellucidum, *FTT* failure to thrive, *STV* small testicle volume, *SS/GR* short stature or growth retardation

**Table 3:** Non- endocrine phenotypes of *SOX3* duplications

Refs	Case	Sex	ID	Myelum	Senses	Speech	Musculo-skeletal	Kidney	Additional findings
[39]	F IV	?	−	Lumbosacral MMC					
[39]	F	F	−	Lumbosacral MMC and myeloschisis					
[40]	III.2	F	−		Hearing impairment	Dyslalia			
[40]	II.2	F	−		Hearing impairment	Dyslalia			Premature aging, Epilepsy, aneurysm
[40]	III.4	F	−		Hearing impairment	Dyslalia			
[31]	III.2	F	+						
[38]	F1	F	−	MMC			Clubfeet, calf muscle atrophy, 3 sacral vertebrae		
[38]	F2	F	−	Lumbosacral MMC			Varus feet		
[38]	F3	F	−	Lumbosacral MMC			Calf muscle hypotrophy	Bilateral kidney hypertrophy	
[32]	I	M	++						Feeding difficulties
[32]	II	M	++						Other complex disabilities
[32]	III	M	−	Lumbral MMC (repaired after birth)					
[32]	IV	M	+						
[32]	V	M	+		Hyposmia, dysgeusia				
[21]		M	−					Right kidney hypoplasia	
[42]	Index	M	−				Madelung deformity of the forearm, hypoplastic tibia and fibula, clubfeet		
[43]	F2	M	−	MMC					
	F3	M	−	MMC					
[44]	C1	M	−						
[44]	C2	M	−		Ocular abnormalities	Raspy voice language delay			
[44]	C3	M	+						
[44]	C4	M	−						
[39]	III.7	M	+			High pitched voice			
[39]	III.1	M							Obesity
[37]		M	+		Ocular dyspraxia				
[45]		M	+		Hyperphagia	Dysarthria			Behavior problems, minor facial anomalies
[46]		M	−						
[47]	A	M	−						
[47]	C	M	−						
[24]	1	M	−						
[24]	2	M	−						
[28]	A II.1	M	−						
[28]	A II.2	M	−						
[28]	B II.1	M	−						
[28]	B II.2	M	−						
[30]		M	−	Spina bifida					
[48]		M	+		Conductive hearing loss			Single kidney	Feeding difficulties
[29]	1	M	+						
[29]	2	M	+						
[29]	3	M	+						
[27]	IV	M	+						
[27]	IV.4	M	+						
[27]	IV.5	M	+						
[27]	II.5	M	+						
[27]	III.3	M	+						
[27]	III.9	M	+						
[31]	III.9	M	+						
[31]	II.6	M	+						
[31]	II.7	M	+						
[31]	II.8	M	+						
[31]	III.3	M	+						
[31]	III.6	M	+						
[31]	III.7	M	+				Postaxial polydactyly of both hands		
[31]	IV.6	M							Truncal obesity and puffy face
Present case		M	−						
Present case		M	−

**Table 4:** Single nucleotide variants, poly alanine tract insertions and deletions *of SOX3* reported in literature and corresponding clinical findings

Refs	Sex	ID	Clinical findings	Affected pituitary hormones	MRI findings	SOX 3 mutation	Functional relevance
Single nucleotide variants							
[34]	1	*Mild ID*		*GH*	*Small AP; EPP*	*c.424C* > *A; p.142T*	*Predicted as disease-causing transcription activation*
[34]	2			*GH*	*APH*	*c.424C* > *A; p.142T*	
[35]	*M1*	*Mild ID*		*GH, THD, LH/FSH*	*AP*	*c.449C* > *A; p.S150Y*	*Predicted as disease-causing*
[35]	*M2*	*Mild ID*		*GH, THD, LH/FSH*	*DP*	*c.449C* > *A; p.S150Y*	
[35]	*M3*	*Mild ID*		*GH, LH/FSH*	*EP*	*c.449C* > *A; p.S150Y*	
[36]	*M*		*Severe SS/GR*	*GH, LH and FSH*	*APH*	*c.14G* > *A; p.R5Q*	*No functional effect, benign likely benign*
[24]						*c.127G* > *A; p.A43T*	*polymorphism*
Poly alanine tract variants							
[25]	*M*		*Short stature*	*GH, TSH, ACTH*	*APH; EPH*	*p.239-245del7*	*Increased transcription activity*
[36]	*F*	*Normal intelligence*	*SS/GR*	*GH, TSH, LH/FSH*	*Enlarged AP; NPP*	*p.243-248del6*	*Transcription activation; Repress β-catenin*
[37]	*M*	*Normal intelligence*	*SS/GR*	*All*	*NA*	*p.A240-241ins7*	*Loss of transcriptional activity; Reduced nuclear transport unable to repress β-catenin*
[37]	*M*	*Learning difficulties*	*SS/GR*	*GH*	*APH; EPP*		
[49]	*M*		*SS/GR*	*GH*	*Normal AP; EPP*	*p.A240-241ins7*	*Loss of transcriptional activity; Reduced nuclear transport unable to repress β-catenin*
[49]	M	*Normal intelligence*		*GH*	*APH; EPP*		
[24]	M	*Normal intelligence*	*SS/GR*	*GH, TSH, LH/FSH, ACTH*	*NA; AHI*	*p.A240-241ins7*	*Loss of transcriptional activity; Reduced nuclear transport unable to repress β-catenin*
[16]	*M*	*Normal intelligence*		*GH, TSH, LH/FSH, ACTH*	*APH; EPP, AHI*		
[16]	*M*		*SS/GR*	*GH, TSH, LH/FSH, ACTH*	*APH; EPP, AHI*		
[33]	M	*X-linked ID*	*SS/GR*	*GH*	*NA*	*p.A234-245ins11*	
[33]	*M*	*Severe ID*	*SS/GR*	*–*	*NA*	*p.240-248del9*	*Transcription activation; Repress β-catenin*

*PA* polyalanine, *ID* intellectual disability, *APH* anterior pituitary hypoplasia, *EPP* ectopic posterior pituitary, *AHI* absent or hypoplastic infundibulum, *AP* absent pituitary gland, *DP* dysplastic pituitary gland, *EP* ectopic pituitary gland, *NA* not available (adapted from Tagaki et al. 2013)

## Discussion

We performed SNP array analysis and WES in a family with unexplained hypopituitarism. SNP array data revealed a 6 Mb duplication of Chromosome X at position Xq26.2-q27.1. The duplication included *GPR101*, a single exon gene that has been associated with X-LAG and acromegaly. *GPR101* encodes an Orphan G-protein Coupled Receptor (GPCR) that is strongly expressed in the hypothalamus in rodents [13–15]. In humans, high expression of *GPR101* is seen during fetal development of the pituitary gland while expression is low in the adult pituitary, suggesting that *GPR101* is predominantly active during proliferation and maturation of the pituitary. Overexpression of *GPR101* leads to increased Growth Hormone-Releasing Hormone (GHRH) expression, which causes hyperplasia of the pituitary and leads to increased GH and IGF-I concentrations [14].

Xq26.3 (micro) duplications including *GPR101* have been described in patients with X-linked acro-gigantism (X-LAG). X-LAG is characterized by early age pediatric-onset gigantism associated with mixed GH-PRL secreting pituitary adenomas, or hyperplasia that leads to GH and IGF-I overexpression resulting in gigantism [14, 16–19]. In 2014, the smallest region of overlap (SRO) was reported, which was shared by all X-LAG patients, and which contains four genes: *CD40LG* (MIM 300386), *ARHGEF6* (MIM 300267), *RBMX* (MIM 300199) and *GPR101* (MIM 300393). Of these four genes, only *GPR101* was overexpressed in pituitary samples of X-LAG patients [16]. Two years later, a smaller duplication including *GPR101* only was reported in a patient with X-LAG, thereby supporting the causative role of *GRP101* [16]. In addition, *GPR101* variants have been identified in pituitary adenoma samples of patients with sporadic acromegaly [16].

The duplication of an acrogigantism gene in the two boys with the phenotypic opposite (hypopituitarism) was unexpected. However, the duplication also included *SOX3,* also a single exon gene, which has been associated with variable degrees of hypopituitarism. *SOX3* belongs to the SOX (SRY-related high mobility group- box) family of transcription factors that is expressed in neuro-epithelial progenitor and stem cells in the earliest stages of embryogenesis [20–23]. SOX3 protein is required for normal development of the brain, pituitary and face in mice and humans. Correct gene dosage of *SOX3* is critical for the development of the hypothalamo-pituitary axis and for cognitive development [24–26].

Duplications including *SOX3* have been associated with variable clinical phenotypes, including X-linked intellectual disability (ID), GHD, X-linked hypopituitarism (XH), SRY-negative 46,XX disorders of sex development (DSD) and neural tube defects (NTD) [27–33]. The severity of the phenotype is not dependent on the size of the duplication. Hypopituitarism is the most frequently reported phenotype among males with *SOX3*, duplication, followed by GHD. Most of the reported cases were of familial origin with transmission of the duplicated region from mother to son. Transmission to females often resulted in a NTD phenotype, and in most cases elective termination of pregnancies. However females with *SOX3* duplications can also have a normal phenotype, which is probably due to non-random X-inactivation of the affected X chromosome. Normal X-inactivation is a random process which is thought to have arisen during the differentiation of mammalian sex chromosomes to achieve an equal dosage of X chromosome genes in females and males (as males only possess a single copy of the X chromosome). Non-random X inactivation might explain the presence or absence of a X-LAG phenotype. In females with *GPR101* duplications, non-random inactivation of the affected allele can lead to a normal phenotype. When inactivation of the affected X does not occur at a high rate, leaving expression of the affected copy, females with *GPR101* duplications do have the X-LAG phenotype. This mechanism is likely also true for females with *SOX3* duplications. These females often have a normal phenotype, due to the non-random inactivation of the affected allele. Only few females with *SOX3* duplications are clinically affected, probably due to the lack of inactivation of the affected allele.

Apart from *SOX3* duplications, *SOX3* single nucleotide substitutions (three point variants and one polymorphism) have also been described. Two variants (p.S150Y and p.142T), predicted as pathogenic, were found in patients with pituitary anomalies with GHD or hypopituitarism with ID [24, 34–36]. Several insertions and deletions found in the first poly-alanine tract of *SOX3* have been described in patients with short stature with IGHD, with and without cognitive impairment [5, 24, 25, 33]

Although rare, large duplications and deletions including both *GPR101* and *SOX3* have previously been reported [28, 30, 31, 37]. A Xq26.1–q27.3 duplication was reported in 2 male patients with hypopituitarism only [28], whereas a deletion of this region was reported in a male patient with panhypopituitarism who also had ID, spina bifida (NTD), and growth retardation [30]. Another Xq26.3–27.3 duplication was reported in a male patient with severe growth retardation, ocular abnormalities, hypotonia, seizures and developmental delay [37]. Hamel et al. reported the largest duplication (Xq24–q27.3) containing *GPR101* and *SOX3* in a male patient with ID, GHD and growth retardation [31].

These data support our current finding that, in duplications containing both *GPR101* and *SOX3,* the GHD phenotype is dominant. This is probably explained by the timing of *GPR101* and *SOX3* expression. SOX proteins are crucial for the patterning and morphogenetic processes occurring in the *early* embryo. During early embryogenesis, cells are organized by tissue patterning. This means that induction of fate-determining genes is spatially controlled to generate patterns for cell differentiation and maturation. *GPR101* is predominantly active during maturation of the pituitary [14], which take place at a later stage. As *SOX3* is affected, and patterning is thus already disturbed, the later possible effects of *GPR101* overexpression in the pituitary might be overruled. We cannot disregard the possibility that dysregulation of other genes in this duplicated region might contribute to the suppression of *GPR101*.

In conclusion We found a 6 Mb duplication of Xq26.2-q27.1 in two brothers with hypopituitarism, which included *GPR101*, a gene associated with the phenotypic opposite: X-linked acrogigantism. Additional analysis showed that the duplication also included *SOX3*, a gene involved in early pituitary organogenesis which is associated with variable degrees of hypopituitarism. Our findings, supported by the literature, show that in duplications containing both *GPR101* and *SOX3*, the GHD phenotype is dominant. This suggests that *GPR101* duplication is overruled when early patterning of the embryonic pituitary is affected due to *SOX3* duplication. The fact that the mother (carrying the same duplication as the two boys) was unaffected, is probably due to non-random X inactivation. Our results, combined with our genotype–phenotype analysis, sheds a new light on the genetic background of both hypopituitarism and gigantism.

## Electronic supplementary material

Below is the link to the electronic supplementary material.

Electronic supplementary material 1 (PNG 75 kb). Supplemental figure 1: Overview of previously described point mutations in GPR101 (3A) and SOX3 (3B). A. Overview of GPR101 gene mutations described to date in literature and corresponding clinical findings. B. Overview of previously described point mutations found in the 1st poly-alanine tract of SOX3 protein with the corresponding clinical findings. Allele frequency in normal population is shown below nucleotide and protein sequence

Electronic supplementary material 2 (PDF 69 kb)

## Abbreviations

ACTH: Adrenocorticotrophic hormone
*ARHGEF6*: Rho guanine nucleotide exchange factor 6
AVP: Vasopressin
*CD40LG*: CD40 ligand
CH: Congenital hypopituitarism
PHD: Pituitary hormone deficiency
IGHD: Isolated growth hormone deficiency
CPHD: Combined pituitary hormone deficiency
FSH: Follicle stimulating hormone
GH: Growth hormone
*GH1*: Growth hormone 1 gene
GHD: Growth hormone deficiency
GHRH(R): Growth hormone releasing hormone (receptor)
*GLI2*: Glioma-associated oncogene family zinc finger 2
GPCR: G protein coupled receptor
*GPR101*: G protein-coupled receptor 101
*HESX1*: HESX homeobox 1
*HHIP*: Hedgehog interacting protein
*HPRT*: Hypoxanthine phosphoribosyltransferase 1
ID: Intellectual disability
*IGF-1*: Gene encoding insulin-like growth factor -1
IGF-I: Insulin-like growth factor -1 (protein)
*IGFBP*: Insulin-like growth factor binding protein
LH: Luteinizing horrmone
*LHX3*: LIM homeobox 3
*LHX4*: LIM homeobox 4
MRI: Magnetic resonance imaging
NTD: Neural tube defect
*OTX2*: Orthodenticle homeobox 2
OXY: Oxytocin
*POU1F1*: POU domain, class 1 transcription factor 1 (PIT1)
PRL: Prolactin
*PROP1*: Prophet of POU1F1
*RBMX*: RNA-binding motif protein, X chromosome
*SOX3*: Sex determining region Y box 3
TSH: Thyroid stimulating hormone
X-LAG: X-linked acromegaly
