# Supplementary figures and images for: Congenital hypopituitarism in two brothers with a duplication of the ‘acrogigantism gene’ GPR101: clinical findings and review of the literature
Source: Pituitary. 2020 Nov 13;24(2):229–41. doi: 10.1007/s11102-020-01101-8 (PMC7966638; doi:10.1007/s11102-020-01101-8)

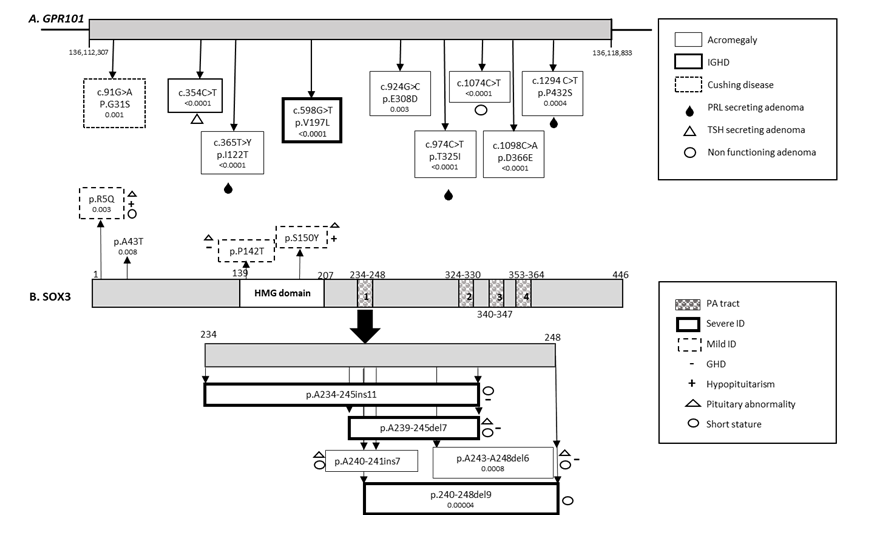

Supplement: Supplementary file 1 — Electronic supplementary material 1 (PNG 75 kb). Supplemental figure 1: Overview of previously described point mutations in GPR101 (3A) and SOX3 (3B). A. Overview of GPR101 gene mutations described to date in literature and corresponding clinical findings. B. Overview of previously described point mutations found in the 1st poly-alanine tract of SOX3 protein with the corresponding clinical findings. Allele frequency in normal population is shown below nucleotide and protein sequence [file 11102_2020_1101_MOESM1_ESM.png]
