# Supplementary material for: Congenital hypopituitarism in two brothers with a duplication of the ‘acrogigantism gene’ GPR101: clinical findings and review of the literature
Source: Pituitary. 2020 Nov 13;24(2):229–41. doi: 10.1007/s11102-020-01101-8 (PMC7966638; doi:10.1007/s11102-020-01101-8)
Supplement: Supplementary file 2 — Electronic supplementary material 2 (PDF 69 kb) [file 11102_2020_1101_MOESM2_ESM.pdf]

Supplemental table 1: 70 genes included in the duplicated region found in the two brothers

chrom	ChromStart	Chrom End	Gene Symbol	Gene Description	Disease association
chrX	133507341	133562822	PHF6	Homo sapiens PHD finger protein 6	Borjeson-Forssman-Lehmann syndrome (BFLS), characterized by cognitive disability, epilepsy, hypogonadism, hypometabolism, obesity, swelling of subcutaneous tissue of the face, narrow palpebral fissures, and large ears.
chrX	133594174	133634698	HPRT1	hypoxanthine phosphoribosyltransferase 1	Mutations in this gene result in Lesch-Nyhan syndrome (a condition characterized by neurological and behavioral abnormalities and the overproduction of uric acid in the body) or gout.
chrX	133674214	133674292	MIR450B	microRNA 450b	
chrX	133674370	133674461	MIR450A1	microRNA 450a-1	
chrX	133674537	133674637	MIR450A2	microRNA 450a-2	
chrX	133675370	133675467	MIR542	microRNA 542	
chrX	133678456	133680653	MIR503HG	MIR503 host gene	Pre-Eclampsia
chrX	133680357	133680428	MIR503	microRNA 503	tumor suppressor
chrX	133680643	133680741	MIR424	Homo sapiens microRNA 424 (MIR424), microRNA.	
chrX	133684053	133694428	LINC00629	long intergenic non-protein coding RNA 629	Gastric cancer
chrX	133699872	133792513	PLAC1	placenta-specific 1	Pre-Eclampsia
chrX	133830061	133830113	Mir_1302	Rfam model RF00951	Recurrent Implantation Failure, male infertility
chrX	133903595	133930385	FAM122B	family with sequence similarity 122B	
chrX	133941222	133988641	FAM122C	family with sequence similarity 122C	
chrX	134021661	134049297	MOSPD1	Homo sapiens motile sperm domain containing 1 (MOSPD1), mRNA.	Short stature and Right ventricle development
chrX	134124967	134126503	SMIM10	small integral membrane protein 10	
chrX	134154533	134156566	FAM127C	family with sequence similarity 127, member C	
chrX	134166332	134167575	FAM127A	family with sequence similarity 127, member A	
chrX	134184962	134186221	FAM127B	family with sequence similarity 127, member B	
chrX	134229014	134232733	LINC00087	long intergenic non-protein coding RNA 87	
chrX	134252881	134254405	LINC00633	s long intergenic non-protein coding RNA 633	
chrX	134254548	134257529	BC061642	uncharacterized protein;	
chrX	134290460	134305751	CXorf48	chromosome X open reading frame 48	chronic myeloid leukemia
chrX	134382887	134477957	DKFZp451F083	Homo sapiens mRNA; cDNA DKFZp451F083 (from clone DKFZp451F083); complete cds.	
chrX	134419722	134429965	ZNF75D	Homo sapiens zinc finger protein 75D (ZNF75D), transcript variant 2, mRNA.	
chrX	134478695	134497338	ZNF449	zinc finger protein 449	
chrX	134540020	134540794	AB062081	cDNA, SINE-R retroposon, isolate:HFB-1.	
chrX	134555867	134560225	LINC00086	long intergenic non-protein coding RNA 86 .	Gastric cancer
chrX	134562092	134562210	5S_rRNA	Rfam model RF00001	
chrX	134654554	134716460	DDX26B	DEAD/H box polypeptide 26B	Autism spectrum disorder
chrX	134847184	134856988	CT45A1	cancer/testis antigen family 45, member A1	
chrX	134883487	134891519	CT45A3	cancer/testis antigen family 45, member A3	
chrX	134928696	134936735	CT45A4	Homo sapiens cancer/testis antigen family 45, member A4 (CT45A4), mRNA.	
chrX	134945650	134953994	CT45A5	cancer/testis antigen family 45, member A5	
chrX	134963214	134971244	CT45A6	cancer/testis antigen family 45, member A6	
chrX	134975784	134995220	SAGE1	sarcoma antigen 1	tumor specific,excellent sources of antigens for cancer immunotherapy.
chrX	135044230	135056134	MMGT1	membrane magnesium transporter 1	
chrX	135067585	135129428	SLC9A6	solute carrier family 9, subfamily A	Mental retardation, X-linked syndromic cognitive disability, Christianson type.

chrom	ChromStart	Chrom End	Gene Symbol	Gene Description	Disease association
chrX	135229558	135293518	FHL1	four and a half LIM domains 1	patients with Emery-Dreifuss muscular dystrophy, reducing body myopathy, Uruguay faciocardiomusculoskeletal syndrome
chrX	135298973	135333738	MAP7D3	MAP7 domain containing 3	
chrX	135570124	135574598	BRS3	bombesin-like receptor 3	
chrX	135579670	135594503	HTATSF1	HIV-1 Tat specific factor 1	
chrX	135614310	135638966	VGLL1	vestigial like 1	Tumor suppressor and cancer
chrX	135633036	135633119	MIR934	microRNA 934	
chrX	135721701	135724588	LINC00892	long intergenic non-protein coding RNA 892	
chrX	135730335	135742549	CD40LG	CD40 ligand	A defect in this gene results in an inability to undergo immunoglobulin class switch and is associated with hyper-IgM syndrome
chrX	135747711	135863503	ARHGEF6	Rac/Cdc42 guanine nucleotide exchange factor (GEF) 6	
chrX	135856157	135856264	U6	Rfam model RF00026 hit found at contig region AL683813.10/545-651	
chrX	135955605	135962939	RBMX	Homo sapiens RNA binding motif protein, X-linked (RBMX), transcript variant 1, mRNA.	X-linked intellectual disability syndrome
chrX	135961357	135961430	SNORD61	Homo sapiens small nucleolar RNA, C/D box 61 (SNORD61), small nuclear RNA.	
chrX	135991553	136075814	AK055694	Homo sapiens cDNA FLJ31132 fis, clone IMR322000953.	
chrX	136112306	136113833	GPR101	G protein-coupled receptor 101	Pituitary adenoma 2, GH-secreting, XLAG
chrX	136648345	136654259	ZIC3	Zic family member 3	Heterotaxy and congenital heart disease
chrX	136770049	136770153	U6	Rfam model RF00026 hit found at contig region AL591398.2/79459-79356	
chrX	137696891	137699799	LINC00889	Homo sapiens long intergenic non-protein coding RNA 889 (LINC00889), non-coding RNA.	
chrX	137713733	138287185	FGF13	fibroblast growth factor 13	Borjeson-Forssman-Lehmann syndrome (BFLS),
chrX	137749871	137749954	MIR504	microRNA 504	
chrX	137794268	137798763	FGF13-AS1	FGF13 antisense RNA 1	
chrX	137922164	137922184	JB158051	Sequence 1454 from Patent EP1777301.	
chrX	138528977	138531132	SRD5A1P1	steroid-5-alpha-reductase, alpha polypeptide 1 pseudogene 1	
chrX	138612894	138645617	F9	Homo sapiens coagulation factor IX (F9), mRNA.	factor IX deficiency, which is a recessive X-linked disorder, also called hemophilia B or Christmas disease.
chrX	138663929	138790381	MCF2	Homo sapiens MCF.2 cell line derived transforming sequence (MCF2), transcript variant 3, mRNA.	biomarker for Borderline personality disorder (BPD)
chrX	138681375	138681459	Mir_548	Rfam model RF01061 hit found at contig region AL033403.1/72536-72619	
chrX	138808504	138914447	ATP11C	Homo sapiens ATPase, class VI, type 11C (ATP11C), transcript variant 1, mRNA.	Congenital hemolytic anemia
chrX	139006306	139006390	MIR505	Homo sapiens microRNA 505 (MIR505), microRNA.	
chrX	139037883	139047677	CXorf66	Homo sapiens chromosome X open reading frame 66 (CXorf66), mRNA.	
chrX	139173825	139175070	LOC389895	Homo sapiens chromosome 16 open reading frame 72-like (LOC389895), mRNA.	
chrX	139533312	139533373	U7	Rfam model RF00066 hit found at contig region AL121875.10/53331-53391	
chrX	139585151	139587225	SOX3	SRY (sex determining region Y)-box 3	associated with X-linked cognitive disability with growth hormone deficiency (GDH), Mental retardation (MR), X-linked hypopituitarism, 46,XX, Disorder of Sex and neural tube defects (NTD)
